# Gluten‐Free Pan Bread Enriched With Microalgae (*Dunaliella salina*) and Encapsulated *Lactobacillus acidophilus*

**DOI:** 10.1002/fsn3.71294

**Published:** 2025-11-26

**Authors:** Seyedeh Fatemeh Ziaziabari, Babak Ghiassi Tarzi, Seyed Mahdi Seyedain‐Ardebili

**Affiliations:** ^1^ Department of Food Science and Technology, Science and Research Branch Islamic Azad University Tehran Iran

**Keywords:** *Dunaliella salina*, encapsulation, gluten‐free bread, *Lactobacillus acidophilus*, probiotic bread

## Abstract

Celiac disease is a chronic intolerance to specific cereal prolamins containing particular oligopeptide sequences, leading to a lifelong intestinal disorder in gluten‐sensitive individuals. The only effective treatment for this condition is adherence to a lifelong gluten‐free diet. This study aimed to develop gluten‐free pan bread using rice and potato flours enriched with *Dunaliella salina* microalgae and *Lactobacillus acidophilus* . The bacterial strain was encapsulated using two different methods with three substances: emulsification/coating with chitosan, sodium caseinate, and whey protein isolate (WPI), followed by spray drying. The encapsulated probiotic bacteria, along with the microalgae, were incorporated into the gluten‐free bread formulation. Farinograph analysis revealed that the addition of *Dunaliella salina* increased water absorption, softening degree, and farinograph quality number, while reducing dough development and stability times. The encapsulation method significantly influenced the pH and acidity of the bread samples (*p* < 0.05), whereas the concentration of the microalgae had no significant effect on these parameters. The highest acidity was observed in the bread containing bacteria encapsulated with sodium caseinate and WPI. Increasing the concentration of *Dunaliella salina* resulted in a decrease in lightness (L*) and an increase in a* and b* values (*p* < 0.05). Encapsulation significantly enhanced probiotic viability in all treated samples compared to the control, with the highest probiotic count found in the sample encapsulated via emulsification/coating with chitosan (*p* < 0.05). Sensory evaluation demonstrated that increasing the microalgae concentration significantly improved the color, appearance, and texture sensorial evaluation scores of the bread, while reducing taste, odor, and overall acceptance. The results indicated that incorporating encapsulated *Lactobacillus acidophilus* with chitosan and 6 g *Dunaliella salina* per 100 g flours substantially enhances the nutritional and technological properties of gluten‐free bread. Furthermore, gluten‐free bread enriched with microalgae can serve as an effective carrier for the probiotic strain *Lactobacillus acidophilus* .

## Graphical abstract

Gluten‐free pan bread was produced using rice–potato flour supplemented with *Dunaliella salina* (3 and 6 g/100 g) and *Lactobacillus acidophilus* (1 g/100 g) encapsulated via chitosan emulsification (A and B) and spray‐dried sodium caseinate (C and D) and WPI (E and F). The formulations yielded distinct loaf structures, where chitosan encapsulation maximized probiotic survival (A and B), and higher (6 g/100 g) microalgae levels (B, D, and F) intensified crumb pigmentation and modified rheological behavior, enhancing technological performance.

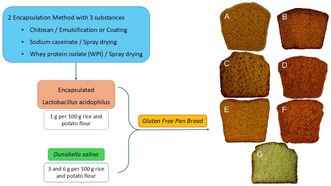

## 1 Introduction

The food industry not only supplies a variety of high‐quality food products with desirable sensory and nutritional properties but also plays a crucial role in addressing the dietary needs of individuals who cannot consume certain products due to medical conditions. Celiac disease and lactose intolerance are among the health concerns that have prompted the development of specialized food products. Celiac disease is a chronic intolerance to specific cereal prolamins containing particular oligopeptide sequences, leading to a lifelong intestinal disorder in gluten‐sensitive individuals. The only effective treatment for this condition is adherence to a lifelong gluten‐free diet, which results in clinical improvements (Biesiekierski 2017; Gallagher et al. 2004; López et al. 2004). Consequently, gluten‐free bakery products must be developed using alternative cereals such as rice (Taromsari and Ghiassi Tarzi 2024). Several studies have explored gluten‐free bread production using rice flour (Choi et al. 2015; Hong and Kweon 2020; Kawamura‐Konishi et al. 2013; Matos and Rosell 2013), a blend of white and black rice flours (Choi et al. 2015), and a combination of rice and black wheat flours (Torbica et al. 2010). These studies demonstrated that rice and potato flour are suitable substitutes for wheat in producing gluten‐free bread with physicochemical and sensory properties comparable to conventional wheat bread.

Given the essential role of wheat bread in supplying vital vitamins and micronutrients, as well as the necessity of eliminating it from the diet of individuals with celiac disease, efforts have been made to enhance the nutritional quality of gluten‐free bread using natural bioactive compounds. Carotenoids, a class of strategic bioactive compounds, have gained significant attention in the food industry due to their numerous health benefits (Camacho et al. 2019). They are naturally synthesized by photosynthetic organisms such as microalgae, which contain substantial amounts of digestible proteins, fiber, vitamins, β‐carotene, iron, and omega‐3 and omega‐6 fatty acids (Camacho et al. 2019).

*Dunaliella salina* , the most commercially significant natural source of β‐carotene, is a unicellular microalga belonging to the Chlorophyceae class. It contains β‐carotene, lycopene, lutein, xanthine, and cryptoxanthin and can biosynthesize and accumulate β‐carotene up to approximately 15% of its dry weight. As a result, it is considered a potential natural anticancer agent (Polle et al. 2020; Widowati et al. 2017). Various microalgae, including *Spirulina platensis*, *Dunaliella salina* , and *Chlorella sorokiniana* , have been incorporated into different food products such as pasta (Bazarnova et al. 2021; El‐Baz et al. 2017; Rodríguez De Marco et al. 2014), bread (Tertychnaya et al. 2020; Yaiche Achour et al. 2014), and cookies (Şahin 2020). Additionally, previous studies have explored the enrichment of gluten‐free bread with *Spirulina platensis* (da Silva Figueira et al. 2011), *Ascophyllum nodosum* (Różyło et al. 2017), *Nannochloropsis gaditana* L2, and *Chlamydomonas* sp. EL5 (Khemiri et al. 2020), as well as *Nannochloropsis gaditana*, *Tetraselmis chuii*, and *Chlorella vulgaris* (Qazi et al. 2022).

Moreover, given the prevalence of digestive disorders among individuals with celiac disease and the necessity of developing functional probiotic products (Oscarsson et al. 2021), incorporating probiotic bacteria into gluten‐free bread could help alleviate these issues. To date, there is a dearth of information regarding the production of gluten‐free bread incorporating both a microalga and a probiotic strain. Therefore, the objective of this research was to develop gluten‐free pan bread enriched with *Dunaliella salina* at varying concentrations while also encapsulating the probiotic strain *L. acidophilus* using different techniques before incorporating it into the bread formulation. The dough samples were analyzed for farinograph properties, and the resulting bread was evaluated for its physicochemical, nutritional, and sensory characteristics.

## 2 Materials and Methods

### 2.1 Production of Probiotic Strain

*L. acidophilus* was cultured in 30 mL of MRS broth and incubated at 37°C for 24–48 h until reaching a concentration of 10^8^ CFU/g, as determined using the 0.5 McFarland standard. Subsequently, the fresh microbial cells were harvested by centrifuging the resulting biomass at 5000 × *g* and 4°C for 15 min. The bacterial pellet was then washed twice with sterile 0.1% peptone water (Mokarram et al. 2009).

### 2.2 Encapsulation With Chitosan

The probiotic strain was encapsulated using the emulsification/coating method with chitosan, as described by Seyedain Ardabili et al. (2016). Briefly, a 100 mL solution containing 3% (w/w) alginate and 2% (w/w) resistant starch (Hi‐maize, Ingredion, USA) was prepared. The microbial suspension (0.1%, w/v) was then added to the mixture along with sunflower oil containing Tween 80. Calcium chloride was introduced while stirring the emulsion, leading to bead formation. These beads were separated by centrifugation (Digicen 21, Orto Alresa, Spain) and stored at 4°C. To apply the chitosan coating, a solution of chitosan (4 g/L) was prepared in acetic acid, and the pH was adjusted to 5.7–6 using sodium hydroxide (NaOH). The prepared beads were immersed in this solution and gently stirred for 40 min. Finally, the coated beads were separated, immersed in peptone water for 1 h, and used for probiotic bread production on the same day.

### 2.3 Encapsulation With Sodium Caseinate

The probiotic bacteria were encapsulated using sodium caseinate following the method described by Zhao et al. (2020). A solution containing 2% (w/w) sodium caseinate and 2% (w/w) sucrose was prepared and mixed with *L. acidophilus* (9 log CFU/g). The mixture was stirred at 400 rpm and 40°C for 30 min using a magnetic stirrer. The pH was then adjusted to 6 using 1 M hydrochloric acid (HCl). Afterward, the mixture was placed in an ice bath for 15 min and subsequently spray‐dried using a Büchi B‐191 spray dryer (Büchi, Germany).

### 2.4 Encapsulation With Whey Protein Isolate (WPI)

A solution of WPI (10%, w/w) was prepared by dissolving WPI in sterile distilled water at room temperature, followed by overnight refrigeration to ensure complete hydration. The pH of the solution was then adjusted to 7 using 1 M NaOH. The microbial suspension was subsequently added to achieve a bacterial concentration of 3–4 × 10^9^ CFU/mL. Finally, the mixture was spray‐dried following the method described by Khem et al. (2016).

### 2.5 Production of Gluten‐Free Bread Enriched With Probiotics and Microalgae

The gluten‐free bread formulation consisted of rice flour (1000 g), potato starch (1000 g), sugar (60 g), salt (40 g), xanthan gum (10 g), margarine (120 g), and water (1710 g), which were thoroughly blended. Subsequently, yeast (60 g) was added to the mixture, and mixing continued at a low speed for 1 min followed by a high speed for 2 min. The dough was then divided into 400 g portions, transferred to a proofer (80% humidity, 4°C, 30 min), and later baked at 220°C–225°C for 25 min to produce gluten‐free pan bread. After baking, the loaves were cooled and packaged in polyethylene bags (Alvarez‐Jubete et al. 2010). The gluten‐free pan bread containing nonencapsulated *L. acidophilus* was designated as the control. To produce probiotic‐enriched bread, 1 g of encapsulated *L. acidophilus* bacteria was incorporated per 100 g of rice and potato flours (Seyedain Ardabili et al. 2016). Similarly, to produce microalgae‐enriched bread, microalgae powder was added at concentrations of 3 and 6 g/100 g based on flour weight (Table 1).

**TABLE 1:** Formulations of gluten‐free pan bread based on flours' weight.

Sample	Abbreviation	Encapsulated *L. acidophilus* (% w/w)	Encapsulating material	Encapsulating method	*Dunaliella salina* (% w/w)
Control	—	1 g/100 g NE	None	None	None
1	CH3M	1 g/100 g	Chitosan	Emulsification/coating	3 g/100 g
2	CH6M	1 g/100 g	Chitosan	Emulsification/coating	6 g/100 g
3	SCA3M	1 g/100 g	Sodium caseinate	Spray‐drying	3 g/100 g
4	SCA6M	1 g/100 g	Sodium caseinate	Spray‐drying	6 g/100 g
5	WPI3M	1 g/100 g	Whey protein isolate (WPI)	Spray‐drying	3 g/100 g
6	WPI6M	1 g/100 g	Whey protein isolate (WPI)	Spray‐drying	6 g/100 g

*Note:* Nonencapsulated bacteria. *L. acidophilus* and *Dunaliella salina* percent weights were calculated based on 100 g of rice and potato flours.

### 2.6 Evaluation of Dough Properties

#### 2.6.1 Rheology

The rheological properties of the dough samples were analyzed using a farinograph (Brabender, Duisburg, Germany) following the method described by Cappelli, Guerrini, et al. (2020). This method was employed to determine the water absorption, dough development time, stability, and degree of softening. In this procedure, a defined amount of flour (typically 300 g on a dry matter basis) was placed into the farinograph bowl, and water was gradually added until the dough consistency reached 500 Brabender Units (BU). The volume of water required to achieve this consistency was recorded as the water absorption of the flour. During mixing, key parameters were derived, including water absorption (the percentage of water required to reach 500 BU), dough development time (the time to attain maximum consistency), dough stability (the duration for which maximum consistency was maintained), and degree of softening (the decrease in consistency after a defined time period). These parameters were used to assess flour quality and to predict dough performance in baking processes. The farinograph test is a standardized and widely adopted method in food industries for characterizing dough rheology.

### 2.7 Microbial Population in Gluten‐Free Bread With Probiotic

Immediately after baking, 10 g of bread was mixed with 100 mL of phosphate buffer (0.1 M, pH = 7) and homogenized in a stomacher at 230 rpm for 10 min. The microbial population was quantified by culturing the sample on MRS agar. After allowing the mixture to settle, 1 mL of the supernatant was inoculated onto the culture medium and incubated at 37°C for 48 h (Zorea and Zorea 2009). To further evaluate the impact of storage, we re‐measured the bacterial population after 7 days of storage at room temperature (25°C) using the method described earlier. This assessment aimed to determine the role of encapsulation in preserving the viability of *Lactobacillus acidophilus* .

### 2.8 Evaluation of Bread Properties

The moisture (AACC 44‐15.02), protein (AACC 46‐12.01, *N* × 6.25), ash (AACC 08‐01.01), fat (AACC 30‐25.01), and crude fiber content (AACC 32‐10.01) of the bread samples were determined based on AACC International Approved Methods (Şahin 2020).

To measure the pH, 10 g of bread crumbs were mixed with 90 mL of distilled water and stirred with a glass rod to ensure thorough mixing. The pH of the mixture was then determined using a pH meter (Jenway 350, UK). Titratable acidity was quantified by titration with 0.1 N NaOH until reaching a pH of 7 (Aplevicz et al. 2013).

#### 2.8.1 Specific Volume and Porosity

The volume, specific volume, and porosity of the gluten‐free bread samples containing probiotics were measured using the rapeseed displacement method. The samples were stored in sealed, sterile polyethylene bags at 25°C, and the tests were conducted after 2 h (Cappelli, Guerrini, et al. 2020).

#### 2.8.2 Assessment of Bread Crust Color Indices

A bread crust slice (6 × 8 cm^2^) was cut and scanned using a high‐resolution scanner (Canon iR‐ADV 4225, Japan) at 600 dpi. The scanned images were analyzed using ImageJ software to extract the color indices L*, a*, and b*. The total color difference (∆*E*) was calculated using the following equation:

$$∆E=\sqrt{{\left(L_{0}^{*}-L^{*}\right)}^{2}+{\left(a_{0}^{*}-a^{*}\right)}^{2}+{\left(b_{0}^{*}-b^{*}\right)}^{2}}$$

where the indices with subscript 0 correspond to the control sample (Zheng et al. 2006).

### 2.9 Sensory Evaluation

The sensory attributes of the bread samples, including color, odor, taste, texture, appearance, and overall acceptance, were evaluated by 10 trained panelists aged 30–40 years. These panelists were specialists in bakery product research and development, as well as sensory evaluation, ensuring a high standard for product development. A five‐point hedonic scale was employed (5 = very good, 1 = very bad) (Şahin 2020). Each panelist was randomly presented with 30 g coded slices of bread, served at room temperature to reduce bias and prevent the formation of sensory patterns. Evaluations took place in a quiet, temperature‐controlled environment. All panelists assessed both the control and six treatment samples at room temperature and were provided with room temperature water to cleanse their palates between tastings, ensuring the removal of any residual flavors. Prior to participation, panelists were informed about the sample ingredients and provided their written consent. Since the participants did not belong to a vulnerable population, formal ethics approval was deemed unnecessary for this evaluation.

### 2.10 Statistical Analysis

Statistical analyses were conducted using SPSS software (version 27, IBM, USA). A one‐way analysis of variance (ANOVA) was utilized to assess the effects of treatment. When significant differences were identified, mean comparisons were carried out using Duncan's multiple range test. All data, including the results from sensory evaluations, were analyzed with ANOVA, and the values are presented as mean ± standard deviation.

## 3 Results and Discussion

### 3.1 Chemical Composition of *Dunaliella salina*

The chemical composition analysis of *Dunaliella salina* is demonstrated in Table 2. These findings are consistent with previous studies investigating the composition of this microalga under stress conditions (El‐Baz et al. 2017; Şahin 2020). According to Şahin (2020), the reported moisture, protein, fat, and total ash contents of *Dunaliella salina* were 6.16%, 22.18%, 3.00%, and 42.81%, respectively. The minor variations observed between these studies may be attributed to differences in the growth environment and the level of stress applied during cultivation (Wu et al. 2020).

**TABLE 2:** Chemical composition of *Dunaliella salina* powder.

Moisture (%)	Protein (%)	Fat (%)	Total ash (%)	Crude fiber (%)
7.55 ± 0.45	24.29 ± 1.07	3.21 ± 0.33	42.96 ± 2.26	7.18 ± 1.28

*Note:* Data are expressed as mean ± standard deviation (*n* = 3). The total composition sums to approximately 85.19%, and the remaining 14.81% corresponds to other components that were not directly analyzed.

### 3.2 Farinograph Properties of Dough Samples

The effects of varying concentrations of *Dunaliella salina* and encapsulated *Lactobacillus acidophilus* on the farinograph properties of dough are summarized in Table 3. The results indicated that the encapsulation method influenced some farinograph indices, as reflected in Table 3. However, variations in the microalgae concentration exhibited a more pronounced impact on these parameters. The incorporation of *Dunaliella salina* at both 3 and 6 g/100 g flours in all samples induced significant changes in water absorption, dough development time, softening degree, and farinograph quality number (*p* < 0.05).

**TABLE 3:** Farinograph properties of gluten‐free bread samples with probiotic.

Sample	Water absorption (%)	Dough development time (min)	Dough stability time (min)	Softening degree of the dough (BU)	Farinograph quality number
Control	65.23 ± 1.63^b^	2.85 ± 0.14^a^	3.29 ± 0.30^a^	52.2 ± 2.63^c^	63.03 ± 1.18^a^
CH3M	66.25 ± 2.27^b^	2.62 ± 0.14^a^	2.33 ± 0.28^b^	86.9 ± 1.39^b^	43.1 ± 1.35^b^
CH6M	71.38 ± 3.76^a^	2.12 ± 0.00^b^	1.35 ± 0.09^c^	108.3 ± 4.71^a^	29.94 ± 0.72^c^
SCA3M	65.89 ± 2.45^b^	2.65 ± 0.12^a^	2.33 ± 0.13^a^	92.3 ± 3.54^b^	44.38 ± 1.60^b^
SCA6M	70.05 ± 4.11^a^	2.14 ± 0.07^b^	1.22 ± 0.11^c^	112.4 ± 4.23^a^	32.02 ± 0.55 ^c^
WPI3M	66.96 ± 1.42^b^	2.62 ± 0.01^a^	2.29 ± 0.00^b^	86.8 ± 2.38^b^	45.11 ± 0.38^b^
WPI6M	71.11 ± 2.33^a^	2.09 ± 0.00^b^	1.41 ± 0.05^c^	111.3 ± 5.87^a^	31.19 ± 0.13^c^

*Note:* Data are expressed as mean ± standard deviation for three replicates. Different letters in the same column indicate statistically significant differences between groups (*p* < 0.05).

The addition of *Dunaliella salina* at 3 g/100 g flours increased water absorption in CH3M, SCA3M and WPI3M compared to the control, although the difference between CH3M, SCA3M and WPI3M was not statistically significant (*p* > 0.05). In contrast, the 6 g/100 g *Dunaliella salina* inclusion in CH6M, SCA6M, and WPI6M led to a significant increase of approximately 6% in water absorption compared to the control (*p* < 0.05). These changes are likely due to the presence of proteins and polysaccharides in *Dunaliella salina* , which contribute to water absorption and enhance the dough's water‐holding capacity. Additionally, the hydrocolloid structure of the microalgae contains hydroxyl groups, which facilitate interactions with water molecules through hydrogen bonding (Ab et al. 2012). Similar findings have been reported for pasta enriched with *Spirulina platensis* (Mostolizadeh et al. 2020), and pasta containing *Dunaliella salina* (El‐Baz et al. 2017).

The inclusion of *Dunaliella salina* led to a reduction in dough development time, which represents the time required to achieve maximum dough consistency. Compared to the control, the addition of *Dunaliella salina* at 3 and 6 g/100 g flours in all samples reduced this parameter by 0.23 and 0.75 min, respectively (*p* > 0.05). Generally, stronger flours require longer dough development times. Despite increasing protein content, the incorporation of *Dunaliella salina* reduced this index. However, since the reduction was not statistically significant, it can be inferred that the microalgae did not negatively affect the technological properties of the final product. El‐Baz et al. (2017) similarly reported that adding *Dunaliella salina* powder to pasta formulation reduced the dough development time from 10.5 to 9 min in the control to 9 min in pasta containing 3% microalgae.

The dough stability time was significantly influenced by *Dunaliella salina* , as it decreased by approximately 1 and 2 min in samples containing 3 and 6 g microalgae per 100 g flours, respectively (*p* < 0.05). Similar findings were reported by El‐Baz et al. (2017), who observed that the addition of *Dunaliella salina* to pasta dough decreased stability time from 11 min in the control to 8 min in samples containing 3% microalgae, indicating a reduction in dough strength.

Furthermore, the softening degree of the dough significantly increased with the addition of *Dunaliella salina* at both 3 and 6 g/100 g flours in samples, especially in SCA6M, which rose by 60.2 farinograph units (BU), respectively (*p* < 0.05). This suggests a softer dough texture after microalgae incorporation.

The farinograph quality number decreased by approximately 18.8 units in CH3M, SCA3M, and WPI3M and approximately 31.9 units in CH6M, SCA6M, and WPI6M from the control (*p* < 0.05). From a technological perspective, this reduction is beneficial, as it can improve the final product's quality. However, these results suggest that *Dunaliella salina* has a negative impact on dough quality. Similar findings have been reported by El‐Baz et al. (2017), who noted a decrease in pasta dough quality with increasing *Dunaliella salina* levels.

### 3.3 Probiotic Bacteria Viability in Bread Samples

The viability of *L. acidophilus* encapsulated using two different methods and three different substances was assessed immediately after production and 7 days later (Table 4). According to the World Health Organization (WHO) and the Food and Drug Administration (FDA), the probiotic count in a food product (e.g., per gram of pan bread) should be at least 10^6^–10^7^ CFU/g at the time of consumption. The results indicated that all bread samples initially contained the recommended probiotic levels. However, only samples CH3M and CH6M maintained this level by the end of the storage period. Consequently, it can be concluded that the emulsification/coating with chitosan was the most effective encapsulation method for preserving bacterial viability during baking and over the 7 days storage period at room temperature, likely due to the formation of a thicker polysaccharide barrier that enhanced thermal resistance and moisture protection (Table 4).

**TABLE 4:** Viability of probiotic bacteria in gluten‐free bread samples during 7 days' storage.

*L. acidophilus* count (10^8^ CFU/g)		
Sample	Time after baking (day)	
	0	7
Control	0.0013 ± 0.00^Ae^	0.00006 ± 0.00^Bc^
CH3M	1.8 ± 0.10^Ab^	0.8 ± 0.10^Ac^
CH6M	2.1 ± 0.20^Aa^	0.8 ± 0.20^Ba^
SCA3M	0.6 ± 0.00^Ad^	0.2 ± 0.00^Bb^
SCA6M	0.8 ± 0.10^Ac^	0.1 ± 0.00^Bc^
WPI3M	0.8 ± 0.00^Ac^	0.3 ± 0.00^Bb^
WPI6M	0.9 ± 0.10^Ac^	0.2 ± 0.00^Bb^

*Note:* Different lowercase letters (a, b, c, d, e) indicate significant differences among means within each column (*p* < 0.05). Different uppercase letters (A, B) indicate significant differences among means within each row (*p* < 0.05). Data are expressed as mean ± standard deviation from three replicates. CH3M: 1 g encapsulated *L. acidophilus* per 100 g flours + Chitosan material + emulsification/coating method +3 g *Dunaliella salina* per 100 g flours, CH6M: 1 g encapsulated *L. acidophilus* per 100 g flours + Chitosan material + emulsification/coating method +6 g *Dunaliella salina* per 100 g flours, SCA3M: 1 g encapsulated *L. acidophilus* per 100 g flours + Sodium caseinate material + spray‐drying method +3 g *Dunaliella salina* per 100 g flours, SCA6M: 1 g encapsulated *L. acidophilus* per 100 g flours + Sodium caseinate material + spray‐drying method +6 g *Dunaliella salina* per 100 g flours, WPI3M: 1 g encapsulated *L. acidophilus* per 100 g flours + Whey protein isolate material + spray‐drying method +3 g *Dunaliella salina* per 100 g flours, WPI6M: 1 g encapsulated *L. acidophilus* per 100 g flours + Whey protein isolate material + spray‐drying method +6 g *Dunaliella salina* per 100 g flours.

Encapsulation improved probiotic bacteria viability in all samples compared to the control (Table 4). The highest probiotic count was observed in CH6M (approximately 1.6 × 10^5^% higher than control), followed by CH3M, both encapsulated via emulsification/coating with chitosan. In contrast, the lowest bacterial viability was recorded in the control sample, which contained nonencapsulated bacteria. Additionally, the viability of bacteria in the WPI3M sample was significantly higher than that in the SCA3M sample, with a difference of approximately 33.3%. Variations in the probiotic count immediately after baking may be attributed to differences in the efficiency of encapsulation methods in forming a protective barrier against environmental stressors such as heat (Rajabi et al. 2015). Arslan‐Tontul (2020) encapsulated *Bifidobacterium bifidum* using various carrier compounds and found that whey protein provided significantly better protection than sodium caseinate in maintaining probiotic viability. Similarly, Seyedain Ardabili et al. (2016) reported that *L. acidophilus* and *L. casei* exhibited the highest viability after baking when encapsulated using an alginate/starch/chitosan coating.

After 7 days of storage, the probiotic count significantly decreased in all samples (*p* < 0.05). In agreement with these findings, Hosseininezhad and Abedfar (2018) reported a significant decline in the microbial population of probiotic bread containing *L. acidophilus* and *Bacillus coagulans* encapsulated with edible starch after 48 h. Likewise, Soares et al. (2019) observed a decrease in the viability of *B. coagulans* in pan bread from 10^6^ to 10^5^ CFU/g after 1 week of storage. The effect of the encapsulation method on bacterial viability followed a similar trend to that observed immediately after baking, with the highest viability recorded in samples CH6M, followed by CH3M, and the lowest in the control sample.

### 3.4 Physicochemical Properties of Bread Samples

The pH of the final product is a crucial factor influencing the viability of probiotics in fermented foods. The acidity of the bread samples ranged between 6.76% and 7.80%, while their pH values were within the range of 5.7–5.9 (Table 5). Similar results have been reported for the acidity and pH of probiotic bread by Soares et al. (2019). It has been established that an approximate pH of 6 is optimal for the growth and viability of probiotic bacteria (Seyedain Ardabili et al. 2016). Therefore, it can be inferred that gluten‐free bread enriched with *Dunaliella salina* serves as a suitable carrier for the probiotic strain *L. acidophilus*.

**TABLE 5:** Physicochemical properties of gluten‐free bread samples with probiotic.

Sample	Titrable acidity (%)	pH	*a*_w_	Moisture (%)	Protein (%)	Ash (%)
Control	6.76 ± 1.29^b^	5.9 ± 0.1^a^	0.96 ± 0.03^a^	34.45 ± 2.19^c^	5.41 ± 0.55^c^	1.16 ± 0.10^c^
CH3M	6.85 ± 0.36^b^	5.9 ± 0.0^a^	0.92 ± 0.00^b^	38.08 ± 1.45^b^	6.17 ± 0.36^b^	2.24 ± 0.07^b^
CH6M	6.83 ± 0.51^b^	5.9 ± 0.0^a^	0.91 ± 0.01^b^	41.33 ± 0.87^a^	6.96 ± 0.32^a^	3.87 ± 0.53^a^
SCA3M	7.80 ± 0.15^a^	5.7 ± 0.1^b^	0.93 ± 0.01^b^	37.64 ± 1.05^b^	6.24 ± 0.14^b^	2.33 ± 0.10 ^b^
SCA6M	7.76 ± 0.11^a^	5.7 ± 0.0^b^	0.92 ± 0.00^b^	40.62 ± 1.61^a^	6.69 ± 0.09^a^	3.81 ± 0.09^a^
WPI3M	7.55 ± 0.43^a^	5.7 ± 0.1^b^	0.93 ± 0.02^b^	37.87 ± 2.01^b^	6.33 ± 0.55^b^	2.23 ± 0.11^b^
WPI6M	7.51 ± 0.19^a^	5.7 ± 0.0^b^	0.92 ± 0.00^b^	42.13 ± 1.39^a^	6.71 ± 0.27^a^	3.59 ± 0.18^a^

*Note:* Data are expressed as mean ± standard deviation in three replicates. Different letters in the same column indicate statistically significant differences between groups (*p* < 0.05).

The control sample did not show significant differences in titratable acidity compared to samples CH3M and CH6M. However, the acidity levels were significantly higher in the other samples (*p* < 0.05). A similar trend was observed in pH levels, as the samples SCA3M, SCA6M, WPI3M, and WPI6M showed significantly lower pH values compared to the control group and the samples CH3M and CH6M. This phenomenon may be attributed to the amphiphilic and water‐soluble nature of sodium caseinate and WPI, which facilitated the rapid release of bacteria. Consequently, this increased acidity and decreased pH were likely due to sugar fermentation by the bacteria (Nag et al. 2011).

Moreover, the degradation of the protective coating during the baking process and subsequent storage likely contributed to a significant decline in bacterial count in these treatments (Jafari et al. 2017; Rajabi et al. 2019). This finding aligns with the results of the bacterial viability test. Increasing the microalgae concentration from 3 to 6 g per 100 g flours did not result in significant changes in pH and acidity (Table 5). In contrast, Khemiri et al. (2020) reported that incorporating microalgae in bread formulation led to a significant increase in bread pH. This discrepancy may be due to differences in gluten‐free bread formulations and the specific type of microalgae used.

### 3.5 Moisture Content and Water Activity

The moisture content of the bread samples ranged from 34.45% to 42.13% (Table 5). The highest moisture content was observed in the sample WPI6M in comparison with control (*p* < 0.05). This increase in moisture content can be attributed to the microalgae's ability to absorb and retain water. These findings are consistent with the results of the farinograph test.

In agreement with the present study, Tertychnaya et al. (2020) reported that increasing the *Dunaliella salina* concentration from 5% to 10% led to a significant rise in the moisture content of bread samples. Similarly, Şahin (2020) observed that cookies containing *Dunaliella salina* powder exhibited higher moisture content than the control.

Conversely, the water activity of gluten‐free bread samples significantly decreased with the addition of the microalgae (*p* < 0.05). The presence of various compounds, including fiber and protein, contributed to the absorption of free water, thereby lowering water activity. These findings align with the results of Sanjari et al. (2018), who reported a similar reduction in water activity in baguette bread enriched with *Spirulina platensis*. Nonetheless, the concentration of microalgae did not significantly influence water activity, as all samples containing 3 and 6 g/100 g of microalgae in the flours showed no notable differences in this parameter (*p* > 0.05).

### 3.6 Protein Content

The protein content of the bread samples ranged from 5.41% to 6.96%, corresponding to sample CH6M and the control, respectively. As expected, enriching the bread with *Dunaliella salina* led to a significant increase in its protein content (*p* < 0.05), as this microalga contains approximately 25% protein.

Previous studies have demonstrated that the incorporation of *Spirulina platensis* into gluten‐free bread increased its protein content by 39% (da Silva Figueira et al. 2011). Similar findings have been reported by Tertychnaya et al. (2020) for gluten‐free bread enriched with *Dunaliella salina* , El‐Baz et al. (2017) for pasta fortified with *Dunaliella salina* , Khemiri et al. (2020) for gluten‐free bread containing *Nannochloropsis gaditana* L2 and *Chlamydomonas* sp. EL5, and Şahin (2020) for cookies supplemented with *Dunaliella salina* .

### 3.7 Ash Content

The ash content of the bread samples ranged from 1.16% to 3.87%. The incorporation of both 3 and 6 g *Dunaliella salina* in 100 g flours in all samples led to a significant increase in ash content (*p* < 0.05), as this microalga contains approximately 43% ash. These findings are consistent with previous research conducted by Khemiri et al. (2020), Şahin (2020), and Yaiche Achour et al. (2014), who also reported an increase in ash content following the enrichment of baked goods with *Dunaliella salina* .

### 3.8 Volume, Specific Volume, and Porosity of Gluten‐Free Bread

**FIGURE 1:**
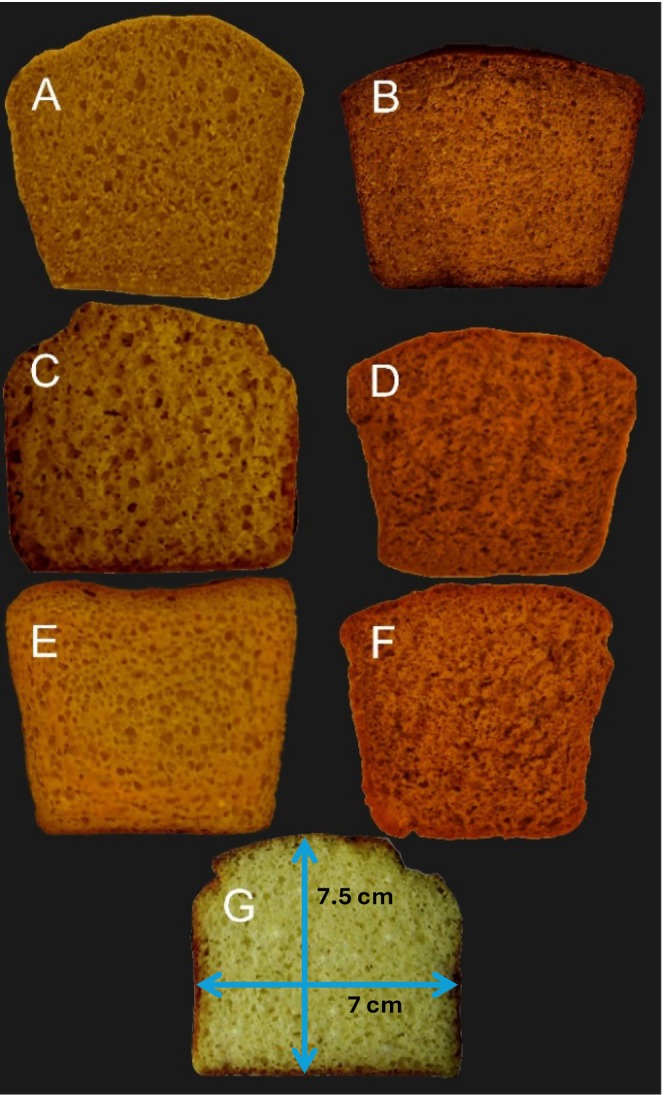
Probiotic gluten‐free bread containing *Dunaliella salina* microalgae. (A) CH3M; (B) CH6M; (C) SCA3M; (D) SCA6M; (E) WPI3M; (F) WPI6M; (G) Control sample.

The texture of bread is affected by several factors, including the protein content, fermentation conditions, and any additives included in the recipe. As illustrated in Table 6 and Figure 1, the volume of the bread significantly increased with the addition of 3 g of *Dunaliella salina* per 100 g of flour, particularly in sample SCA3M. However, at 6 g, the volume decreased to a level even lower than that of the control sample (*p* < 0.05). Similar findings have been reported, where the incorporation of *Spirulina platensis* in bread formulations resulted in notable increases in both volume and specific volume (Yaiche Achour et al. 2014).

**TABLE 6:** Volume related properties of gluten‐free bread samples with probiotic.

Sample	Volume (cm^3^)	Specific volume (cm^3^/g)	Porosity (%)
Control	163 ± 12^b^	1.91 ± 0.10^b^	56.19 ± 2.11^b^
CH3M	171 ± 9^a^	2.23 ± 0.05^a^	59.30 ± 3.21^a^
CH6M	157 ± 7^c^	1.76 ± 0.11^c^	51.65 ± 2.20^c^
SCA3M	173 ± 11^a^	2.25 ± 0.32^a^	59.67 ± 1.05^a^
SCA6M	155 ± 4^c^	2.73 ± 0.30^c^	51.49 ± 2.21^c^
WPI3M	169 ± 7^a^	2.18 ± 0.21^a^	60.11 ± 1.11^a^
WPI6M	156 ± 3^c^	1.75 ± 0.07^c^	51.91 ± 1.19^c^

*Note:* Data are expressed as mean ± standard deviation in three replicates. Different letters in the same column indicate statistically significant differences between groups (*p* < 0.05).

A similar trend was observed for specific volume, where the highest value (2.73 cm^3^/g) was recorded for SCA6M, whereas the lowest (1.75 cm^3^/g) corresponded to the WPI6M sample. The decline in both volume and specific volume with the higher microalgae concentration (6 g/100 g flours) in CH6M, SCA6M, and WPI6M may be attributed to the increased water absorption, which consequently enhanced dough adhesiveness. This, in turn, negatively impacted the elasticity and gas‐holding capacity of the dough (Sanjari et al. 2018). Minh (2014) similarly reported that incorporating *Spirulina platensis* up to 1.25% increases the volume of sweet bread, whereas at 1.5%, a decline in volume was observed. da Silva Figueira et al. (2011) also found that enriching gluten‐free bread with 2%–5% *Spirulina platensis* led to a 22% reduction in bread volume at the 4% concentration level.

As indicated in Table 6, the highest porosity (60.11%) was observed in WPI3M, while the lowest (51.49%) was associated with SCA6M. Similar to volume and specific volume, porosity significantly increased with the 3 g microalgae addition in 100 g flours compared to the control, but decreased at 6 g/100 g (*p* < 0.05). These findings are consistent with Sanjari et al. (2018).

Due to the absence of a gluten network in gluten‐free bread, carbon dioxide produced during fermentation is more readily released from the dough matrix, resulting in a lower porosity than that of gluten‐containing bread (Cappelli, Oliva, and Cini 2020). Any modifications that improve gas retention in gluten‐free dough ultimately lead to an increase in porosity in the final product. As previously discussed, *Dunaliella salina* positively influenced dough structure, enhancing carbon dioxide retention, which contributed to higher volume and specific volume. These findings align with Tertychnaya et al. (2020), who reported that increasing *Dunaliella salina* concentrations in bread led to significant increases in volume, specific volume, and porosity.

### 3.9 Assessment of Bread Crust Color Indices

Color is a crucial sensory attribute that directly influences the overall acceptance of food products. The color indices of the gluten‐free bread samples are presented in Table 7. The results indicated that increasing the *Dunaliella salina* concentration from 3 to 6 g/100 g in flours in all samples significantly reduced the brightness (L*) of the samples. The a* value, representing the red‐green spectrum, was significantly increased by the addition of the microalgae (*p* < 0.05). Moreover, *Dunaliella salina* led to a significant increase in the b* value (yellow‐blue spectrum) as the microalgae concentration increased (*p* < 0.05). These findings align with previous studies, which reported that the incorporation of microalgae into bakery formulations induces significant alterations in color indices (El‐Baz et al. 2017; Mosaddegh et al. 2019; da Silva Figueira et al. 2011).

**TABLE 7:** Color indices of gluten‐free bread samples with probiotic.

Sample	L*	a*	b*
Control	68.75 ± 2.49^a^	0.88 ± 0.25^a^	8.59 ± 0.69^c^
CH3M	63.12 ± 3.05^b^	3.19 ± 0.11^b^	10.42 ± 1.00^b^
CH6M	59.83 ± 2.11^c^	6.34 ± 0.83^c^	13.84 ± 2.06^a^
SCA3M	64.09 ± 4.25^b^	3.34 ± 0.36^b^	9.96 ± 1.67^b^
SCA6M	58.05 ± 2.00^c^	6.57 ± 1.30^c^	13.42 ± 1.14^a^
WPI3M	62.34 ± 4.72^b^	3.05 ± 0.09^b^	9.79 ± 0.85^b^
WPI6M	57.19 ± 1.13^c^	6.61 ± 0.96^c^	13.66 ± 1.22^a^

*Note:* Data are expressed as mean ± standard deviation in three replicates. Different letters in the same column indicate statistically significant differences between groups (*p* < 0.05).

### 3.10 Sensory Analysis

As shown in Table 8, all sensory attributes were significantly influenced by the addition of *Dunaliella salina* (*p* < 0.05). The incorporation of the microalgae improved the bread's color, likely due to the intensification of browning during baking, a desirable reaction in bread production. This finding aligns with previous studies reporting the significant effects of microalgae on the sensory properties of bakery products (El‐Baz et al. 2017; Şahin 2020; Yaiche Achour et al. 2014).

**TABLE 8:** Sensory analysis of gluten‐free bread with probiotic enriched with *Dunaliella salina* .

Sample	Sensory properties					
	Color	Odor	Taste	Appearance	Texture	Overall acceptance
Control	4.29 ± 0.30^a^	5.00 ± 0.00^a^	4.86 ± 0.12^a^	4.35 ± 0.23^a^	4.26 ± 0.37^a^	4.57 ± 0.17^a^
CH3M	4.59 ± 0.37^a^	4.62 ± 0.39^a^	4.53 ± 0.11^a^	4.53 ± 0.28^ab^	4.49 ± 0.22^a^	4.18 ± 0.26^a^
CH6M	5.00 ± 0.00^b^	3.75 ± 0.48^b^	3.62 ± 0.34^b^	5.00 ± 0.00^c^	5.00 ± 0.00^b^	3.67 ± 0.11^b^
SCA3M	4.55 ± 0.11 ^a^	4.65 ± 0.23^a^	4.77 ± 0.26^a^	4.64 ± 0.21^ab^	4.53 ± 0.08^a^	4.31 ± 0.37^a^
SCA6M	5.00 ± 0.00^b^	3.52 ± 0.60^b^	3.52 ± 0.53^b^	5.00 ± 0.00^c^	5.00 ± 0.00^b^	3.60 ± 0.16^b^
WPI3M	4.53 ± 0.16^a^	4.62 ± 0.37^a^	4.57 ± 0.21^a^	4.66 ± 0.10^b^	4.45 ± 0.17^a^	4.29 ± 0.13^a^
WPI6M	5.00 ± 0.00^b^	3.67 ± 0.43^b^	3.41 ± 0.20^b^	5.00 ± 0.00^c^	5.00 ± 0.00^b^	3.53 ± 0.26^b^

*Note:* Data are expressed as mean ± standard deviation in three replicates. Different letters in the same column indicate statistically significant differences between groups (*p* < 0.05).

However, the inclusion of 3 and 6 g *Dunaliella salina* in 100 g flour in all samples led to a significant decrease in the odor scores of the samples (*p* < 0.05). Similarly, El‐Baz et al. (2017) and Marcinkowska‐Lesiak et al. (2018) reported that the addition of *Dunaliella salina* in pasta and *Spirulina platensis* in cookies significantly reduced odor scores compared to control samples. The taste scores followed a similar trend, further supporting previous findings (El‐Baz et al. 2017; Yaiche Achour et al. 2014).

Conversely, the appearance scores of the bread samples significantly increased with higher microalgae concentrations, particularly in CH6M, SCA6M, and WPI6M samples. Consumers often associate red hues with enhanced taste perception, which may have contributed to this improvement. Additionally, the inclusion of *Dunaliella salina* at both levels significantly enhanced texture scores (*p* < 0.05). As demonstrated in the staling measurement test, the hardness of the samples decreased with microalgae addition, leading to improved softness, a change positively perceived by panelists. The highest texture score was recorded for CH6M, SCA6M, and WPI6M samples.

Despite these positive effects on color, appearance, and texture, the overall acceptance of the bread samples significantly declined at both 3 and 6 g/100 g microalgae concentrations (*p* < 0.05). This reduction was likely due to the adverse impact on odor and taste, the primary sensory attributes influencing consumer preference. Similar findings have been reported in previous studies (El‐Baz et al. 2017; Şahin 2020; da Silva Figueira et al. 2011; Yaiche Achour et al. 2014).

## 4 Conclusion

This study examined the production of gluten‐free bread enriched with *Dunaliella salina* and the probiotic *Lactobacillus acidophilus* . The results showed that adding 3 and 6 g microalgae in 100 g rice and potato flour positively influenced the bread's physicochemical, nutritional, and sensory properties. Protein (6.96%) and ash content (3.87%) increased, while higher moisture and lower water activity contributed to improved shelf life. All samples had a softer texture, and staling occurred more slowly. The color and appearance improved with the addition of higher concentrations of microalgae (CH6M, SCA6M, and WPI6M), but this also led to a decrease in overall acceptance. In contrast, all samples with 3 g of microalgae per 100 g of flour provided a better balance. Encapsulation of probiotics with chitosan enhanced their survival during baking, and pH remained within the optimal range. The CH6M sample exhibited the most favorable results in terms of nutritional composition, physicochemical properties, and probiotic bacterial viability; however, it showed comparatively lower scores in sensory attributes than the CH3M sample. This research suggests that gluten‐free bread enriched with *Dunaliella salina* and probiotics encapsulated with chitosan could serve as a functional product for celiac patients. Optimizing the formulation can lead to high‐quality bread with enhanced nutritional value.

## Author Contributions

**Seyedeh Fatemeh Ziaziabari:** methodology (lead); resources (lead); writing – original draft (lead); writing – review and editing (equal). **Babak Ghiassi Tarzi:** methodology (supporting); resources (supporting); supervision (lead); writing – original draft (supporting); writing – review and editing (equal). **Seyed Mahdi Seyedain‐Ardebili:** supervision (supporting).

## Funding

The authors have nothing to report.

## Ethics Statement

All panelists were briefed about the ingredients in preparation for the sensory evaluation, and written consent was subsequently obtained. As the panelists were not a vulnerable population, formal ethics approval was not required for this evaluation.

## Conflicts of Interest

The authors declare no conflicts of interest.

## Data Availability

The data that support the findings of this study are available from the corresponding author upon reasonable request.
