# Correction to “Gluten‐Free Pan Bread Enriched With Microalgae (*Dunaliella salina*) and Encapsulated *Lactobacillus acidophilus*”

**DOI:** 10.1002/fsn3.71434

**Published:** 2026-01-09

Ziaziabari S. F. Ghiassi Tarzi B. Seyedain‐Ardebili S. M. 2025 “Gluten‐Free Pan Bread Enriched With Microalgae (*Dunaliella salina*) and Encapsulated *Lactobacillus acidophilus*.” *Food Science & Nutrition* 13 no. 12 e71294: https://doi.org/10.1002/fsn3.71294.

The affiliation for all three authors was **incorrectly** listed as “Department of Food Science and Technology, Science and Research Branch, Islamic Azad University, Tehran, Iran.”

This should be **corrected** to “Department of Food Science and Technology, SR.C., Islamic Azad University, Tehran, Iran.”

In accordance with a recently issued and officially enforced directive from Islamic Azad University, the institutional affiliation format must be revised. This update is mandatory and directly affects the final thesis defense of the corresponding graduate student involved in this work.

We hereby confirm that no other changes are requested or required. The scientific content, authorship, acknowledgments, and all other elements of the published article remain entirely unchanged.

We sincerely apologize for this error.

